# Differential Expression of the Circadian Clock in Maternal and Embryonic Tissues of Mice

**DOI:** 10.1371/journal.pone.0009855

**Published:** 2010-03-24

**Authors:** Hamid Dolatshad, Andrew J. Cary, Fred C. Davis

**Affiliations:** Department of Biology, Northeastern University, Boston, Massachusetts, United States of America; Sapienza University of Rome, Italy

## Abstract

**Background:**

Molecular feedback loops involving transcription and translation and several key genes are at the core of circadian regulatory cycles affecting cellular pathways and metabolism. These cycles are active in most adult animal cells but little is known about their expression or influence during development.

**Methodology/Principal Findings:**

To determine if circadian cycles are active during mammalian development we measured the expression of key circadian genes during embryogenesis in mice using quantitative real-time RT-PCR. All of the genes examined were expressed in whole embryos beginning at the earliest age examined, embryonic day 10. In contrast to adult tissues, circadian variation was absent for all genes at all of the embryonic ages examined in either whole embryos or individual tissues. Using a bioluminescent fusion protein that tracks translation of the circadian gene, *per2*, we also analyzed protein levels. Similar to mRNA, a protein rhythm was observed in adult tissue but not in embryonic tissues collected *in-vivo*. In contrast, when tissues were placed in culture for the continuous assay of bioluminescence, rhythms were observed in embryonic (E18) tissues. We found that placing embryonic tissues in culture set the timing (phase) of these rhythms, suggesting the importance of a synchronizing signal for the expression of circadian cycles in developing tissues.

**Conclusions/Significance:**

These results show that embryonic tissues express key circadian genes and have the capacity to express active circadian regulatory cycles. In vivo, circadian cycles are not expressed in embryonic tissues as they are in adult tissues. Individual cells might express oscillations, but are not synchronized until later in development.

## Introduction

The coordination of events in time has long been recognized as a salient feature of animal development [1], [2], [3]. A kind of timing, widespread in all groups of organisms, is circadian timing. Endogenously generated 24-hour rhythms give organisms the ability to anticipate predictable environmental change and provide a framework for temporal coordination both within and among individuals. To explore the possible interaction between development and circadian timing, we undertook to determine whether circadian timing is a feature of mammalian embryonic development. We assessed circadian timing in mouse embryos and compared the findings to circadian timing expressed in maternal tissues.

Circadian rhythms are a ubiquitous property of living systems. They are expressed in organisms from bacteria to humans and regulate processes from gene expression to behavior. In animal cells, circadian rhythms arise from transcription/translation feedback loops involving several essential circadian regulatory genes (CRGs). These loops interact with a number of other molecules and processes forming the circadian regulatory cycles (CRCs) which in turn integrate with other cellular pathways ranging from the cell cycle to signal transduction. Circadian rhythms are normally entrained to environmental cycles, most commonly the light/dark cycle. In some animals, such as Zebrafish or Drosophila light interacts directly with tissues throughout the body [4], [5]. In mammals, entrainment by light is mediated by the retina and a central circadian pacemaker in the hypothalamus, the suprachiasmatic nucleus (SCN). The SCN entrains rhythms in other tissues via neural, endocrine and/or other physiological systems in ways that are not fully understood.

In mammals, CRGs include the bHLH-PAS transcriptional regulators, *clock*, *npas2*, and *bmal1* that activate genes for the PAS domain proteins, *per1*, *per2*, *cry1*, and *cry2* via E-box enhancers. These proteins accumulate in the cytoplasm and translocate to the nucleus where PER-CRY heterodimers inhibit their own transcription. The *clock* (or *npas2*)/*bmal1* complex also activates *Rev-erbα*, which represses transcription of *bmal1* through inhibition of the ROR family of transcriptional activators acting at ROR response elements (RORE) [6]. *Clock*/*bmal1* also regulates transcription of albumin D element-binding protein (*Dbp*)[7], a member of the PAR leucine zipper transcription factor family that in turn controls the expression of other regulatory genes[8]. The robust oscillations in these core transcriptional regulators propagate within the cell to drive widespread regulation of transcription [9], ultimately affecting most aspects of cellular activity from metabolism to stress responses [10], [11]. 10–15% of transcription in adult tissues is under circadian regulation [12], [13], [14], [15], [16]

Loss of function in CRGs disrupts circadian rhythms and affects the health of animals [17]. In some instances, developmental processes, such as cell proliferation and differentiation are affected. The proliferation of mouse embryonic fibroblasts and the regeneration of adult liver are disrupted by CRG inactivation [18], [19] as is the differentiation of adipocytes and bone formation [20], [21]. In Zebrafish embryos, cell proliferation is regulated by circadian rhythms [22] and a daily rhythm in fetal growth was reported in rats [23]. These and other results suggest an influence of circadian timing on development. Consistent with this are reports that lower weight fetuses were observed in mice kept under non-24 hour light/dark cycle [24] [25]. In addition, *Per1* and *Per2* knockout mice showed higher number of implantation sites in comparison to WT mice and lower number of delivered offspring, suggesting embryonic losses [26]. *Bmal1* knockout mice have been reported to show delayed embryo development or early embryo loss [27]. Clock mutant mice show higher number of mid gestation fetal reabsorption [28] and lower number of pups born when mice are kept under continuous darkness [29]. These studies do not adequately discriminate between circadian clock disruptions acting through maternal deficiencies or through direct effects on embryonic development. Nevertheless, it is possible that the pervasive influence of CRCs extends to development, affecting fundamental processes such as proliferation, growth and differentiation. Surprisingly little is known about the expression of CRCs during mammalian development.

Previous research on the ontogeny of mammalian circadian rhythms focused on the structural and functional development of the hypothalamic pacemaker, the SCN. Much of this work was performed before the discovery of CRGs. The major finding of this early work was that even in rodents with relatively short gestations, a circadian clock, probably in the SCN, becomes functional before birth and is entrained by maternal rhythms. The consensus was and still is that as soon as SCN neurons become post mitotic and the nucleus forms, the SCN begins to generate oscillations even though synapses are rare and the normal input for entrainment from the retina has not yet developed. [30], [31]. Consistent with this earlier work, CRGs are expressed in the SCN before birth. In rats and hamsters, however, in situ hybridization failed to detect prenatal rhythms [32], [33], [34]. Other studies suggest that molecular rhythms are entrained by maternal rhythms just before birth even if the rhythms are difficult to measure until after birth [35], [36], [37], [38]. In transgenic rats with a luciferase reporter driven by the *per1* promoter, fetal SCN and liver cultured a day before birth expressed rhythms that appeared to be influenced by the timing of the mother's feeding schedule during gestation, suggesting the presence of active and entrainable CRCs in those tissues before birth. It is likely that in at least some portion of SCN cells CRCs become active between completion of SCN neurogenesis and birth, but maturation of the SCN as a robust pacemaker appears to occur after birth [32].

The discovery of CRCs in most adult tissues extends developmental questions to tissues other than the SCN and to earlier times in development. It is possible that embryonic tissues express CRCs that are entrained by the same signals that entrain those in maternal peripheral tissues. mRNAs of several CRGs (*per1*, *cry1* and *bmal1*) have been detected in preimplantation mouse embryos (before E4), but it was not determined whether the mRNAs were rhythmic [39]. A more recent study of preimplantation mouse embryos also reported CRGs immediately after fertilization both *in-vitro* and *in-vivo*, especially *clock* and *bmal1* (probably of maternal origin initially), but rhythms were not seen over the first four days up to the blastocyst stage [40]. In utero imaging of transgenic rat embryos revealed bioluminescence driven by the *per1* promoter from E8 to E22. Expression steadily increased from E10 onward and there was a suggestion of day night differences. A clear rhythm could not, however, be established [41]. In another study, CRGs were measured by PCR in fetal and newborn rat liver, and only *Rev-erbα* showed a rhythm on E20. It was not until sometime between postnatal days 20 and 30 that all of the measured CRGs showed rhythms [42]. In contrast, more recent studies using transgenic rats with a luciferase reporter of *per1* promoter activity observed rhythms *in-vitro* from livers collected the day before birth or the first day after [43], [44]. Earlier ages were not examined. Thus the limited studies on the initial expression of CRCs in mammalian peripheral tissues are inconsistent; *in-vitro* measurements indicate the presence of rhythms at earlier ages than do measurements of tissues collected *in-vivo*. The goal of the present study was to evaluate the presence or absence of a circadian clock in embryonic and maternal tissues using both *in-vitro* and *in-vivo* measurements in mice. To assess CRC activity we measured components of the core transcription/translation feedback loops, several CRGs and one protein (PER2). While some other type of CRC involving different mechanisms could exist in adult or embryonic cells, in this study CRC refers to the regulatory cycles known to depend on the key components we measured.

## Methods

### Animals and Care

Wild-type C57Bl6 mice were purchased from Jackson Laboratories (Bar Harbor, ME) or Charles River Breeding Laboratories (Wilmington, MA). Animals were maintained on a 12:12 hour Light/Dark cycle with food and water available *ad libitum*. All experiments were preformed according to protocols approved by Northeastern University's Internal Animal Care and Use Committee. *mPer2::luc* knockin mice [45]were obtained from Mary Harrington, with the permission of J. Takahashi, and were bred in-house.

For timed pregnancies, females were paired with males for the first two hours of the light period ZT0-ZT2 and plug positive mice were separated. The day of plug detection was considered to be embryonic day 0 (E0). To produce pregnant mice at the same stage of development but with opposite circadian phases, mice were mated as above and kept in the same conditions until 4.5 days after fertilization. By shifting the light:dark cycles mice were separated into two groups, one phase advanced and the other phase delayed. Wheel-running activity records of the mothers showed that by days 9–11 the two groups were entrained to opposite cycles. This produced two groups of pregnant mice with embryos at the same stage of development but with maternal circadian rhythms entrained to opposite light/dark cycles.

### Tissue Harvests

Mice were euthanized by cervical dislocation for adults or decapitation for embryos. Whole embryos or tissue pieces were harvested at various circadian times, frozen on dry ice and stored at –80°C until use. For each time point three pregnant mice were sacrificed and tissues from the mother and fetuses were excised. In some cases hearts and kidneys were excised from previously frozen embryos in phosphate buffered saline (PBS) solution pH 7.4. Heart and kidney samples from 30 day old mice of mixed sex were used as a control for these tissues.

### RNA isolation

Previously frozen tissue samples were ground with a disposable plastic pestle in micro-centrifuge tubes in the presence of 500 µl Triazol (Invitrogen). To each sample was added 100 µl chloroform, mixed, centrifuged at 4°C, and the aqueous supernatant was mixed with similar volume of 70% ethanol and loaded on a SV Total RNA Isolation System (Promega) RNA binding column and processed according to directions. RNA quality was checked by agarose gel electrophoresis and RNA quantification was carried out using Nanodrop 1000 (Nanodrop). Approximately 2 µg of total RNA was reverse transcribed using the Superscript III First Strand Synthesis System (Invitrogen) and resultant cDNAs were stored at –20°C until used.

### RT PCR

The relative expression of CRGs was carried out using Real Time reverse transcriptase-Polymerase chain reaction (RT-PCR) in an Applied Biosystems 7000 Real Time System. Each reaction contained 0.2 ng/µl of cDNA (assuming 100% efficiency of the reverse transcription reaction) in 2x Taqman Universal PCR Master mix (Applied Biosystems) combined with primer and probe each at 100 nM concentrations. Primers and probes used were: *Period1-* Forward- GACCTTGGCCACACTGCAGTA

Reverse- CTCCAGACTCCACTGCTGGTAA, Probe- 6FAM-5′-ATTCCTGGTTAGCCTGAACCTGCTTGACA -BHQ1a (adapted from [46]). *Period2-* Forward- CGGATGCTCGTGGAATCTTCC, Reverse- GGTTGTGCTCTGCCTCTGTC, Probe- 6FAM -5′-CACTCACCCCAGCCCTGATGATGCCT-TAMRA-3′(from [29]). *Bmal1-*

Forward- CCCACAGCATGGACAGCAT, Reverse- CTGGAATGCCTGGGACAGTG, Probe- 6FAM-5′-CTGCCCTCTGGAGAAGGTGGCCA-TAMRA-3′. *Cryptochrome1-*Forward- CAGCAGCTTTCCCGGTACAG, Reverse- GACATTCTCTCCAGGAGCATAGC, Probe- 6FAM-5′-CTAGGTCTTCTCGCCTCGGTCCCTTCTAAC-TAMRA-3. Rev-erb alpha- Forward- AACAGTCTACGGCAAGGCAAC, Reverse- GCAGGCGTGAAGCTCATAGA, Probe- 6FAM-5′-CCGGACTGTGCAGGAGATCTGGGAAGA-TAMRA-3′. *Dbp-* Forward- CATGAGACTTTTGACCCTCGGA, Reverse- CATTGTTCTTGTACCTCCGGCT, Probe- 6FAM-5′-CCAGGTGCCTGAGGAACAGAAGGATGA-BHQ1a-3.

*Gapdh-* Forward- AATGTGTCCGTCGTGGATCTG, Reverse- CAACCTGGTCCTCAGTGTAGC, Probe- 6JOE- 5′-CCGCCTGGAGAAACCTGCCAAGTATGATGA-BHQ1a-3′. *Clock-* Forward- GCTCACGAAAGTCATCTCACAC, Reverse- TTATGGACTGACTGCTGAAGGAC, Probe- 6FAM 5′-CTCAGACCCTTCCTCCACACCGACAAAGAT -BHQ1a-3′.

PCR reactions were run using the standard ABI program, 50°C for 2 min, 95°C for 10 min followed by 40 cycles of 94°C for 15 sec and 60°C for 1 min. PCR reactions contained both an experimental primer probe set as well as the reference primer-probe set for *gapdh* multiplexed in the same reaction [47]. Each primer probe set was standardized prior to use confirming that the efficiency of the reaction for each primer-probe set was 2 allowing for the use of ΔΔCT processing of data. Each data point is the average of three independent samples each assayed with three technical ‘plate’ replicates. Data were tested for significance by either one-way ANOVA or t-test, using SPSS software (Chicago, Il.)

### Cultured tissue rhythms

Tissue samples were excised using sterile technique and placed in chilled Hanks Buffered Saline Solution (HBSS). Approximately 1 mm square tissue pieces were cut and placed in 35 mm petri dishes containing 1.2 ml freshly prepared Luciferase Culture Medium composed of 1.2 ml of Dulbecco's Modified Eagles Medium (Sigma) with 10 mM HEPES pH 7.2, 2% B27 (Gibco), 25 units penicillin, 25 µg streptomycin and 0.1 mM beetle luciferin (Molecular Imaging Products) [48]. Plates were sealed with sterile silicone grease (Fisher) and placed in the Lumicycle (Actimetrics) to record light emissions for at least 5 days. To estimate periods of the expressed rhythms, the best fit sine curve was determined for the 2–5th day interval in culture using the Lumicycle Analysis Software (Actimetrics). Phase was estimated as the peak of the sine wave in the second day, either in relation to the time of dissection or maternal time. The phases were analyzed and graphically displayed using Oriana software (Oriana 3.02, Kovach computing services, UK). An average phase for a group was determined by vector addition and the length of the average vector ® represents the degree of clustering among phases. The probability that a distribution of phases was uniform (random) was determined by the Rayleigh test. A probability (P)<0.05 was taken to indicate significant clustering [49].

### *In-vivo* protein rhythms

Previously frozen liver samples were homogenized with a disposable plastic pestle in a microcentrifuge tube in Luciferase Glo-Lysis Buffer (Promega) at 4°C. Samples were then centrifuged at 4°C and the supernatant retained. Total protein was determined using a Bradford assay, and aliquots were frozen at –80°C till use. Extracts were assayed by combining approximately 300 µg total protein in 100 µl Glo-Lysis buffer with 100 µl One-Glo Luciferase Assay system (Promega), placed in 35 mm Petri dish, sealed with sterile silicone grease and placed in the Lumicycle (Actimetrics) at 37°C. Luminescence was recorded for 75 seconds within 1–5 minutes of substrate addition to reactions and standardized to total protein amounts to derive specific activity.

## Results

### Circadian regulatory genes (CRGs) are expressed throughout post-implanted embryonic development

**Figure 1:**
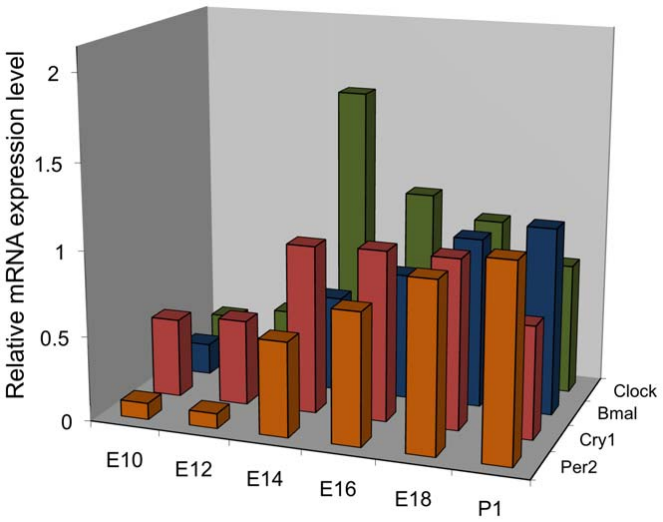
Survey of *Per2*, *Bmal1*, *Cry1* and *Clock* mRNA expression in whole embryos from embryonic day 10 (E10) to postnatal day 1 (P1). Pregnant mice were fed *adlib* and kept on a 12 hr:12 hr Light:Dark cycle. Samples (3–4 embryos per age) were collected at ZT = 5 (lights on = ZT0). The level of each mRNA was measured using quantitative real-time RT-PCR and normalized to *gapdh*.

As a first step towards assaying circadian regulatory cycles (CRCs) in mouse embryos we surveyed the expression of CRGs during post-implantation development. Whole embryos or pups were collected every other day at ZT5 between embryonic day 10 (E10) and post-natal day 1 (P1). Using real time RT-PCR, the transcriptional expression of four CRGs (*Period2*, *Cryptochrome1*, *Bmal1* and *Clock*) were evaluated. CRG mRNA expression levels were determined relative to a control gene, *gapdh*. All four genes were expressed at the mRNA level at all ages (Figure 1). The expression levels of each of the CRG mRNAs were lower during embryonic days 10 and 12 and progressively increased with age thereafter.

### Circadian regulatory cycles (CRCs) are not expressed in post-implanted mouse embryos

**Figure 2:**
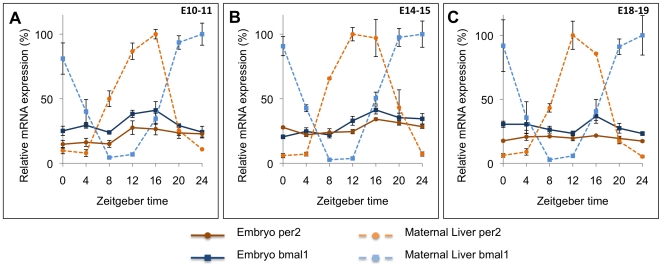
Twenty-four-hour expression profiles of *Per2* and *Bmal1* mRNA in whole embryos and maternal liver during embryogenesis. Whole embryos were collected every 4 hours for 24 hours on E10-E11 (A), E14-E15 (B) and E18-E19 (C) and mRNA was measured using quantitative real-time RT-PCR. The 0 and 24 hour time points were repeated, independent measures of the same time of day. Maternal livers demonstrated robust variation in both *Per2* and *Bmal1* at all ages (P<0.0001), consistent with the rhythms expected for these genes. The mRNA of whole embryos failed to show a clear rhythm in either *Per2* or *Bmal1* at E10-E11 and E18-E19. Low but statistically significant fluctuations were observed for *Per2* and *Bmal1* at E14-E15 (p<0.01). RNA levels were normalized to the control gene, *gapdh*. Symbols represent the mean ± standard error of the mean (SEM) of three biological replicates. The maximum maternal liver RNA for each stage of gestation was set to 100 and the rest of maternal and embryonic samples are presented relative to that maximum.

In order to determine the circadian expression of CRG mRNAs in post-implanted embryos, samples were collected at early (E10-11), mid (E14-15) and late (E18-19) stages of post-implantation development. The expression levels of six mRNAs (*Bmal1*, *Per1*, *Per2*, *Cry1*, *Dbp* and *Rev-erbα*) were measured and compared to maternal liver samples collected at the same time points (Figures 2 and S1). In all genes studied, maternal liver samples showed significant rhythmic expression of the CRGs (P<0.0001). In contrast, no robust rhythms were evident in the embryos.

The embryo samples at E10-11 did not exhibit significant (P>0.01) fluctuations in expression of *Bmal1*, *Cry1*, *Per2 or Rev-erbα* over the 24 hour period (Figures 2 and S1). The remaining two CRGs examined, *Per1* and *Dbp* showed statistically significant variation in abundance (P = 0.007 and P = 0.001 respectively), but the magnitudes of variation were much lower than those in maternal liver samples taken at the same time points.

Embryos collected between E14 and E15 showed small but significant variation in expression of *Bmal1* (P = 0.001) and *Per2* (P = 0.002) (Figure 2). Both genes had their highest level of expression around ZT16 and their lowest between ZT4-8. While the differences in mRNA levels are significant, the amplitudes were much lower than in maternal liver (3–5 fold). In addition, the pattern of *Bmal1* expression was similar to that of *Per2* in the embryo while the maternal liver showed anti-phase expression, consistent with the circadian rhythms described by others [50]. *Per1*, *Cry1*, *Dbp* and *Rev-erbα* expression did not show significant (P>0.05) variation in daily expression (Figure S1). In contrast, maternal liver presented a clear, significant daily rhythm in all genes studied (P<0.0001)(Figures 2 and S1).

Embryos from late stages of gestation (E18-19) did not show significant (P>0.02) variation in the daily expression of any CRGs (*Bmal1*, *Per1*, *Per2*, *Cry1*, *Dbp* and *Rev-erbα*). Although the variation in *Cry1* appeared to be high (Figure S1), the variance among samples was high and differences across time of day were not significant (P = 0.173). Similar to other ages, all mRNAs studied in maternal liver samples showed significant rhythmic expression (P<0.002)(Figures 2 and S1).

### Circadian regulatory cycles (CRCs) are not expressed in embryonic tissues (heart, kidney and liver) when sampled *in-vivo*

**Figure 3:**
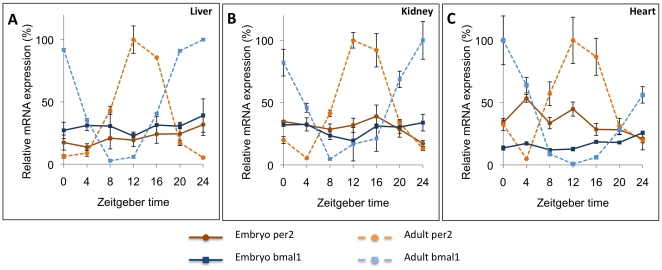
Twenty-four-hour expression profiles of *Per2* and *Bmal1* mRNA in embryonic (E18-E19) and adult tissues. mRNA levels of embryonic liver (A), kidney (B) and heart (C) collected every 4 hours, for 24 hours and mRNA was measured using quantitative real-time RT-PCR. Embryonic tissues showed little variation, especially in comparison to the robust changes seen in adult tissues. Adult liver is represented by the same maternal data shown in Figure 2C and by adult kidney and heart from 30-day old mice. RNA levels were normalized to the control gene, *gapdh*. Symbols represent the mean ± standard error of the mean (SEM) of three biological replicates. For each tissue the maximum adult value was set to 100 and the rest of adult and embryonic samples are presented relative to that maximum.

One explanation for the lack of robust rhythmicity in the E10-11, E14-15 and E18-19 whole embryos is that the individual tissues and organs of the embryos were rhythmic but out of phase with each other. To examine this possibility, the 24-hour mRNA expression profiles of *Bmal1* and *Per2* were determined in heart, kidney and liver from E18-E19 embryos. Similar to the whole embryo, expression of *Bmal1* and *Per2* in embryonic liver and kidney did not show significant variation over 24 hours (P>0.05) while expression in the same tissues from adults showed highly significant variation (P<0.0001) (Figure 3). Although the embryonic heart showed significant fluctuation in both *Bmal1* and *Per2* mRNA expression (P<0.01), *Per2* showed a double peak, at ZT4 and ZT12 with low amplitudes in comparison to adult heart. The *Bmal1* expression in fetal heart increased steadily during the 24 hour period measured, suggesting a developmental trend rather than a rhythm (The repeated time points of ZT0 and ZT24 were significantly different, P = 0.0001).

### Circadian oscillations are expressed in embryonic tissues (heart, kidney and liver) when measured *in-vitro*

**Figure 4:**
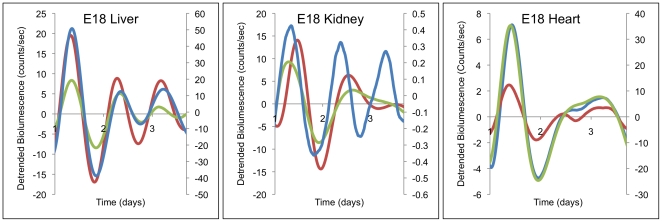
Circadian rhythms expressed by embryonic heart, kidney and liver *in- vitro*. *Per2::luc* embryonic heart, kidney and liver placed in culture show circadian oscillations in light emission with average periods of 24.36, 25.39 and 22.25. The first day of recording is not shown due to transient activity. Data were detrended as described in methods. Tissues from the same embryo are indicated by the same color line.

As a general approach to the development of circadian rhythms, it is advantageous to evaluate rhythms (or their absence) in embryonic or fetal tissues in isolation from maternal rhythms. By doing so, the autonomous origin of rhythms can be established. For this reason, we measured the expression of one CRG, *Per2*, *in-vitro* using *mPer2::luc* transgenic mice, which have a luciferase reporter gene fused to the C-terminal end of the original *Period2* (*Per2*) gene. Surprisingly and in contrast to our real time RT-PCR data, the bioluminescent emission from E18 tissues showed circadian rhythmicity for at least 5 days (Figure 4). The average period for liver, kidney and heart were 22.41, 25.65 and 24.60 hours respectively.

### Circadian oscillations in embryonic liver measured *in-vitro* are set by the culturing procedure

*In-vivo* (RT-PCR) and *in-vitro* (bioluminescence) measurements of *mPer2* expression in fetal liver suggest different conclusions about the presence or absence of active CRCs in fetal tissue. In particular, *in-vivo* measurements showed no evidence of mRNA rhythms while the *in-vitro* cultures of similar tissues were able to express rhythms in mPER2 fusion protein. A possible explanation for this is that placing tissue in culture initiates rhythms that were not expressed *in-vivo*. If this occurs, it is expected that the phase of *in-vitro* rhythms would be determine by the time of the culturing procedure. If phase is instead related to maternal rhythm, this would be strong evidence that the rhythms were actually on-going *in-vivo* before the procedure and had been entrained by maternal rhythms. Two experiments were carried out to determine if the rhythm observed *in-vitro* is set by the culturing procedure.

**Figure 5:**
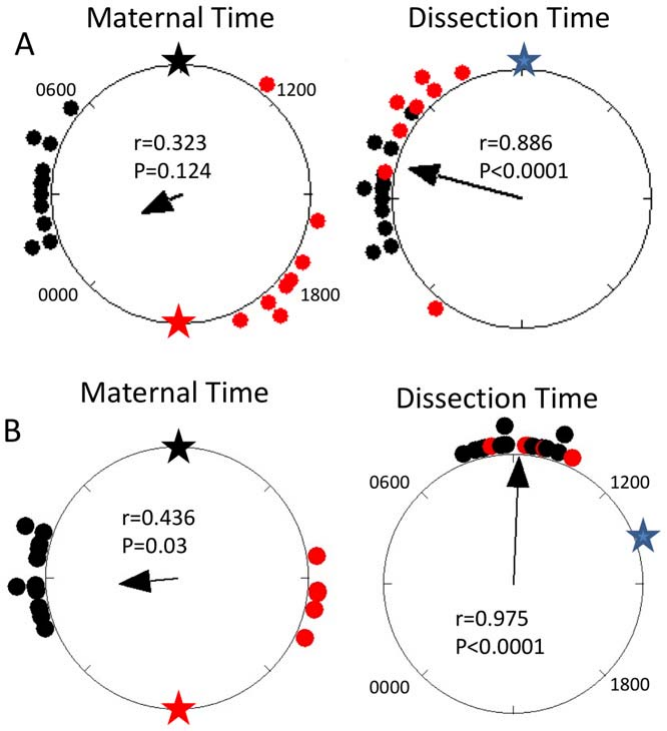
Circular phase plots of embryonic liver rhythms *in vitro* from two experiments showing that *in vitro* phase is set by the explantation procedure. Livers were collected from *Per2::luc* mice and the relative levels of PER2 protein were measured by the recorded light emissions. Phases of *in vitro* rhythms were determined as described in Methods. The large circles represent the second 24 hours in culture and each small circle represents the phase of peak luminescence of an individual sample. In both experiments the same data are plotted relative to two different references, Maternal Time and Dissection Time. The phases of embryonic liver rhythms were consistently clustered relative to dissection time and not relative to maternal time. A. Livers were collected at two times of day, 12 hours apart, ZT12 (E15.5) and ZT0 (E16). Maternal Time corresponds to clock time since all mothers were entrained to the same light:dark cycle with lights on at 0900 (ZT0) and off at 2100 (ZT12). Red circles represent tissues collected at ZT12 (red star) and black circles represent those collected at ZT0 (black star). For Dissection Time phases are plotted relative to the time of dissection (blue star) regardless of the time of day when dissection was done. B. Two groups of mothers were entrained to different light:dark cycles 12 hours apart (see Fig. S3) and livers were collected at one time of day (1400) and at one embryonic age (E15.25). Because mothers were entrained to different ligh:dark cycles Maternal Time does not correspond to clock time. The red symbols represent embryo samples from mothers with lights off at 1400 and black circles represent those from mothers with lights off at 0200. Because dissections were done at the same time of day for both groups (1400, blue star), Dissection Time corrresponds to clock time. The dissections occurred at different times relative to Maternal Time, ZT0 (black star) or ZT12 (red star). The arrow inside each large circle indicates the average phase of all samples. The arrow's length and the r value indicate the degree of synchrony. P is the probability that the distribution of phases is significantly different from uniform (Rayleigh Test). Each experimental group included samples from at least two pregnant mice.

The first experiment involved collecting embryonic liver samples at two times of day, 12 hours apart (0900 and 2100 hr). These were also different ages, approximately E15.5 and E16 (red and black stars respectively in Figure 5A). The samples were immediately placed in lumicycle medium and bioluminescence was measured in the lumicycle. The time of peak bioluminescence on the second day *in-vitro* was determined to represent the phase of the PER2 rhythm. Phases plotted relative to clock time (and light/dark cycle or maternal time) showed two clusters that were about 12 hours apart, matching the difference in dissection times (Figure 5A). When the data were plotted in relation to the dissection time, set as zero (blue star), then the phases of rhythms expressed by samples collected at either time point clustered together suggesting that the tissue rhythms were set by the culturing procedure (Figure 5).

In the second experiment, two groups of female mice were kept on the same light/dark cycle and mated at the same time of day. After mating and after the estimated age of implantation, the groups were exposed to different shifts of the light/dark cycle. For one group the light/dark cycle was phase advanced over two days, E5 to E7, and the other was phase delayed. Between E7 and E15.25 the groups were kept on opposite light/dark cycles. Embryonic liver samples were collected at E15.25 for all animals. While the embryos were at the same developmental stage, they were collected 12 hours apart relative to maternal circadian rhythms and the light/dark cycle (Figure S3). Phases plotted in relation to maternal rhythms showed clusters that were 12 hours apart (Figure 5B). In contrast, when data were plotted in relation to the tissue collection time (Figure 5B), all samples clustered around one time. As with the previous experiment, these results provide no evidence that tissue rhythms were present and entrained *in-vivo* prior to dissection. Instead, the results show that *in-vitro* rhythms are set and possibly initiated by the culturing procedure. Therefore, these results are consistent with the conclusion from RT-PCR measurements that CRCs are not expressed at the tissue level in embryos *in-vivo*.

### Acute luciferase measurements show rhythms in adult tissues but not in embryonic tissues

**Figure 6:**
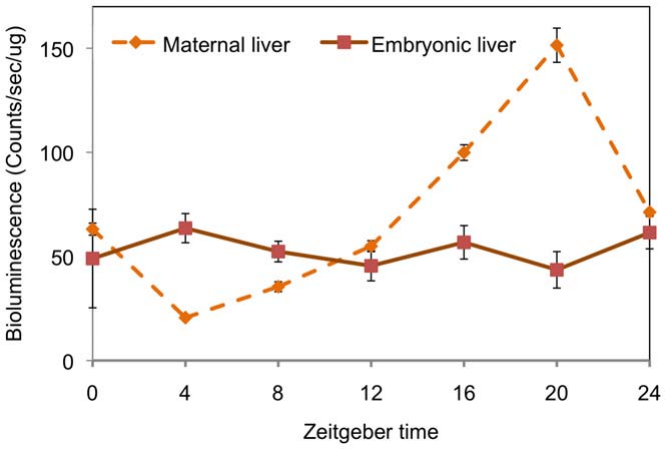
Acute luciferase activity in embryonic and maternal liver. *Per2::luc* mice were used to make protein extracts from maternal and embryonic liver samples every four hours for 24 hours, during E18-19. Luciferase activity was measured as described in Methods. Maternal tissues showed a distinct peak and trough in PER2 levels, at ZT20 and ZT4. In contrast, embryonic liver showed no significant differences during the 24-hour period. Symbols represent the mean ± standard error of the mean (SEM) of three embryos per time point and three technical replicates from a single mother.

The preceding experiments indicated a lack of *in-vivo* rhythms in whole embryos and individual tissues at the mRNA level, yet found a robust rhythm in the same tissues cultured *in-vitro*. It is possible that a rhythm in PER2 protein also exists *in-vivo* even though an mRNA rhythm could not be detected [51]. To determine if there is a rhythm in PER2 protein in embryonic liver *in-vivo* we did an acute assay of luciferase activity in protein extracts made from embryonic liver samples collected every four hours for 24 hours from E18 embryos. Maternal liver samples were collected at the same times. Acute luciferase activity was determined using the lumicycle. A robust circadian oscillation of luciferase activity/PER2 abundance was observed in the maternal liver (P<0.0001) (Figure 6). The maternal PER2 protein levels peaked at ZT20 and the trough was at ZT4. The Per2 mRNA expression peaked around ZT12-16 and the trough was around ZT0-4. From these data, the lag between the mRNA and protein production seems to be around 4–8 hours [52]. In contrast to maternal liver, no significant 24-hour variation was seen in the E18-19 embryonic liver samples (P = 0.818). These results indicate that the different conclusions of the previous *in-vivo* and *in-vitro* measurements, specifically the absence or presence of a rhythm, were not due to the different endpoints used, mRNA versus protein. Instead the circadian rhythm observed in embryonic liver placed in culture may not accurately represent the state of the tissue *in-vivo* prior to dissection.

## Discussion

The regulation of cellular physiology by a circadian regulatory cycle (CRC) is widespread in the tissues of adult mammals. Ultimately, CRC activity in cells also influences systems physiology and behavior. To determine if embryonic development is also influenced by CRC activity, it is first necessary to determine the ontogeny of this regulatory mechanism. The present study found that circadian regulatory genes (CRGs), are expressed in mouse embryos at all ages examined (E10 to E18). At three embryonic ages (E10, E14, and E18) we sampled whole embryos at different times of day every four hours but were unable to detect circadian variation in mRNA accumulation (RT-PCR) or in PER2 protein levels (acute luciferase measurements). A required condition for the measurement of a rhythm in this type of protocol (independent samples collected at different times of day) is that the rhythms in each sample, if present, are synchronized. A population rhythm would not be measured if the samples are not synchronized. The absence of a population rhythm in whole embryos, as observed here, was, however, likely due to the absence of rhythms in individual whole embryos. Individual embryo rhythms would require that a significant portion of tissues and cells within each embryo were rhythmic and synchronized. This hypothetical within embryo synchrony would likely require an external signal such as maternal rhythms. Such a signal would, however, also produce synchrony among embryos and it therefore would have been possible to detect a rhythm by sampling the population if rhythms were in fact present in each embryo.

The absence of circadian rhythms in whole embryos does not exclude the possibility that individual tissues or cells within an embryo express rhythms. Even if entrained by an external signal such as maternal rhythms, different tissues could be entrained with different timings of mRNA peaks and troughs (i.e., different phases). If so, then when assayed together individual tissue rhythms might be obscured. Although the phases of most peripheral rhythms are similar in adults [45], [53], [54], this could be different in embryos. Alternatively, or in addition, if only a few tissues are rhythmic, the rhythm could be hard to detect when assayed with a majority of non-rhythmic tissues. This would certainly be true for the suprachiasmatic nucleus if it expresses rhythmicity during prenatal development as reported[35]. To address these possibilities, we measured CRGs in individual embryonic tissues, heart, kidney and liver. These tissues show robust rhythms in adults. Even in these individual tissues, rhythms were undetectable at embryonic ages. This result has three possible explanations. A particular tissue could express a rhythm (facilitated by coupling mechanisms internal to the tissue) but is not entrained to a common external signal such as maternal rhythms. In this case, the rhythm is unlikely to be detected by sampling different fetuses at different times of day. There is little evidence for strong coupling signals within a tissue, even in adults, making this possibility unlikely. Alternatively, tissue level rhythms could be absent, either because individual cells within the tissue are rhythmic but unsynchronized or because individual cells lack rhythms. A lack of tissue level rhythms, regardless of the explanation, demonstrates a clear difference between embryonic and maternal tissues with respect to circadian regulation.

In six instances (out of 30 possible) statistically significant time of day variation was seen in whole embryos (E14-15, *bmal1* and *per2* and E10-11 *per1* and *Dbp*) and in a specific tissue (heart, E18-E19, *bmal1* and *per2*). These variations were much lower than the rhythms observed in maternal tissues and the patterns were unusual (see Results). We cannot exclude the possibility that there are low amplitude CRCs in embryonic tissues with distinctive profiles, but we do not consider occasional statistical significance to be strong evidence for circadian rhythms.

The explanation that embryonic tissues do not express rhythms because individual cells within the tissue are rhythmic but unsynchronized requires that individual cells do not receive or are insensitive to synchronizing signals. In addition, this hypothesis suggests that while individual cells express a CRC, there is no tissue level function that requires synchrony among cells and the resulting tissue level rhythm. It is likely that there are abundant and robust potential signals from maternal rhythms but that the CRCs in embryonic cells, if expressed, are insensitive to them. Although melatonin is a likely signal for maternal entrainment of CRCs in the fetal suprachiasmatic nucleus (SCN) [55], [56], the lack of melatonin in the C57Bl6 strain of mice [57] is unlikely to be the reason for the absence of CRG rhythms in embryonic peripheral tissues; rhythms were also not detected in a strain of mice (C3H) that does produce melatonin (Figure S2).

If individual cells express CRCs, a possible contribution to within tissue asynchrony unique to growing tissue is interactions between the CRC and cell cycle regulatory mechanisms that reset the timing of the CRC [58]. It is possible that synchrony among cells would occur *in-vivo* if not for the disruptive effects of cell division. *In-vitro*, inhibition of the cell cycle increases the robustness of circadian rhythms [59].

In contrast to the absence of tissue-level rhythms in samples collected from embryos *in-vivo*, we observed rhythms in PER2 expression in embryonic heart, kidney and liver (E18) placed in culture for measurement by a bioluminescent reporter. It is unlikely that the measurement of protein rather than mRNA accounts for the different results; we also did not detect a rhythm when PER2 was measured by bioluminescence acutely in tissue samples collected *in-vivo* (Figure 6). Although continuous measurements from a single piece of tissue (as used for *in-vitro* analysis) might be a more sensitive assay of variation than is the analysis of multiple samples, the latter approach easily detected circadian variation in maternal and other adult tissues. Embryonic tissues are clearly distinct from adult tissues in the expression of CRCs.

The expression of a PER2::LUC rhythm in fetal tissues *in-vitro* is consistent with the possibility that cells within an embryonic tissue are rhythmic but unsynchronized *in-vivo*. Previous measurements of circadian rhythms from cells *in-vitro* indicate that the absence of a rhythm in a population of cells is due to a lack of synchrony among cells rather than the absence of rhythms in each cell [58], [60], [61]. A variety of treatments such as fresh medium or the addition of serum causes synchrony among cells, thereby inducing a population rhythm. [58], [61], [62]. A particular stimulus synchronizes a population of cells by bringing the CRC in each cell to approximately the same phase. Consequently the resulting population rhythm will have a characteristic phase relationship to the stimulus. We found that the rhythms expressed *in-vitro* by embryonic tissues had the same phase relative to the time of the culturing procedure regardless of the time of day or time in the mother's circadian cycle when the procedure was performed. This is the expected result if CRCs within the cells are synchronized by the procedure. While it is possible the procedure initiated CRCs in individual cells, there is no precedence for this in previous studies of cells or tissues *in-vitro*. When zebrafish develop in the absence of an external synchronizing signal (the light/dark cycle), individual cells begin to express a CRC but they are not synchronized across the embryo. Exposure to even a single light pulse synchronizes the CRCs [63].

By E18, cells of embryonic mouse tissues are capable of expressing a CRC and this can be measured at the tissue level *in-vitro*. It is not known, however, when cells first express a CRC *in-vivo* and when they become sufficiently synchronized to produce a measurable tissue-level rhythm. The present results indicate that synchrony does not occur during prenatal development despite exposure to maternal rhythms. Published reports of *in-vivo* CRG mRNA levels indicate that rhythms in CRGs similar to those of adults are not present in rat heart and liver until after postnatal day 20 although some CRGs show 24-hour variation as early as postnatal day 2 [42], [64]. Only one CRG, *rev-erba*, showed 24-hour variation in fetal rat liver (E20) [42], consistent with the lack of rhythms in E18-19 fetal mouse liver reported here. The implications of rhythms in some CRGs but not others in the same tissue at some ages, as reported elsewhere [42], are presently unclear.

We were able to measure CRGs at all embryonic ages examined (E10-E18), and it is likely that CRG mRNA's are present even earlier. For example, CRGs have been measured in pre-implanted embryos [39], [65] [40], [66] but circadian rhythms have not yet been reported. A knock-down of one CRG (*cry1*) in oocytes by inhibitory RNA led to slower completion of meiosis suggesting a regulatory role for *cry1* unrelated to circadian rhythms [40]. This might be the case for other CRGs at other times in development as well. For example, lipogenesis and adipocyte differentiation in mice requires the CRG, *bmal1*, but there is no evidence that these processes also require active CRCs [21]. While CRGs that are transcriptional regulators might have roles during cellular differentiation independent of participation in active CRCs, it is also possible that CRCs are necessarily uncoupled from cellular processes during development. For example, mutations of CRGs affect cell proliferation during liver regeneration [18], proliferation in primary fibroblast culture [19], and progression of the hair follicle cycle [67], but similar mutations do not have obvious affects on growth during embryonic development [68] [69] [70].

In summary, the present results indicate that embryonic mouse tissues do not express CRCs even though they express CRGs and have the potential to express a CRC *in-vitro*. Embryonic tissues appear poised to express CRCs but for some reason do not. It is possible that individual cells within embryonic tissues express a CRC but are not synchronized by maternal rhythms or by signals intrinsic to the tissues. When placed in culture, however, the cells could become synchronized, creating a tissue level rhythm and possibly reinforcing the oscillations in individual cells. This raises interesting questions about how embryonic tissues are shielded from synchronizing perturbations *in-vivo*. Specific mechanisms, in the placenta for example, could facilitate this. Further examination of CRC expression in developing tissues could provide insight into the normal mechanisms of peripheral tissue entrainment in adults. It is possible that CRCs are not expressed even at the level of individual cells in embryonic tissues. The technology required to assess the status of individual cells *in-vivo* is beyond the scope of the present study. The absence of active CRCs in embryonic mouse tissues, as found here, does not exclude the expression of robust CRCs in tissues during prenatal development in mammals with longer gestations, such as humans.

## Supporting Information

Figure S1 Twenty-four-hour expression profiles of Cry1, Per1, Dbp and Rev-erbα mRNA in whole embryos and maternal liver during embryogenesis measured by quantitative real-time RT-PCR. Whole embryos were collected every 4 hours for 24 hours on E10-E11 (A), E14-E15 (B) and E18-E19 (C) with the 0 and 24 hour time points representing repeated, independent measures of the same time of day. Maternal livers (broken lines) demonstrated robust (P<0.001) variation over 24 hours. With the exception of Dbp and Per1 at E10-E11, the embryonic mRNAs did not show significant variation at P = 0.01. RNA levels were normalized to the control gene, gapdh. Symbols represent the mean ± standard error of the mean (SEM) of three biological replicates. The maximum maternal liver RNA for each stage of gestation was set to 100 and the rest of maternal and embryonic samples are presented relative to that maximum.
(0.82 MB TIF)

Figure S2 Expression of Per2 and Bmal1 mRNA in embryonic (E18-E19) and maternal tissues of melatonin positive mice (C3H). mRNA levels in embryonic liver (A) collected every 4 hours for 24 hours and measured using quantitative real-time RT-PCR showed low variation, especially in comparison to maternal liver. The maximum maternal liver mRNA was set to 100 and the rest of maternal and embryonic samples are presented relative to that maximum. In kidney and heart, levels of Per2 and Bmal1 mRNA from embryonic tissues were not significantly different at ZT0 and ZT12 (P>0.4, n = 3), while maternal tissues showed significant differences at these times (P<0.006, n = 3) (t-test). RNA levels were normalized to the control gene, gapdh. Error bars indicate standard error of the means.
(0.59 MB TIF)

Figure S3 Entrainment of pregnant mice to opposite light:dark cycles. The wheel-running activity records of two pregnant mice are shown. Each line of the actograms is 24 hours of recording. Mice were paired with males just before recording was started (double arrow). Between days 5 and 7 of the records the light:dark cycles were shifted, an advance shift for the mouse on the top and a delay shift for the mouse on the bottom. When embryo tissues were collected on the last day of the records (star), the pregnant mice were fully entrained to opposite cycles. Thus the collection of embryo tissues occurred at one age but at two different times within the mothers' circadian cycles.
(0.62 MB TIF)
