# Supplementary figures and images for: Differential Expression of the Circadian Clock in Maternal and Embryonic Tissues of Mice
Source: PLoS One. 2010 Mar 24;5(3):e9855. doi: 10.1371/journal.pone.0009855 (PMC2844431; doi:10.1371/journal.pone.0009855)

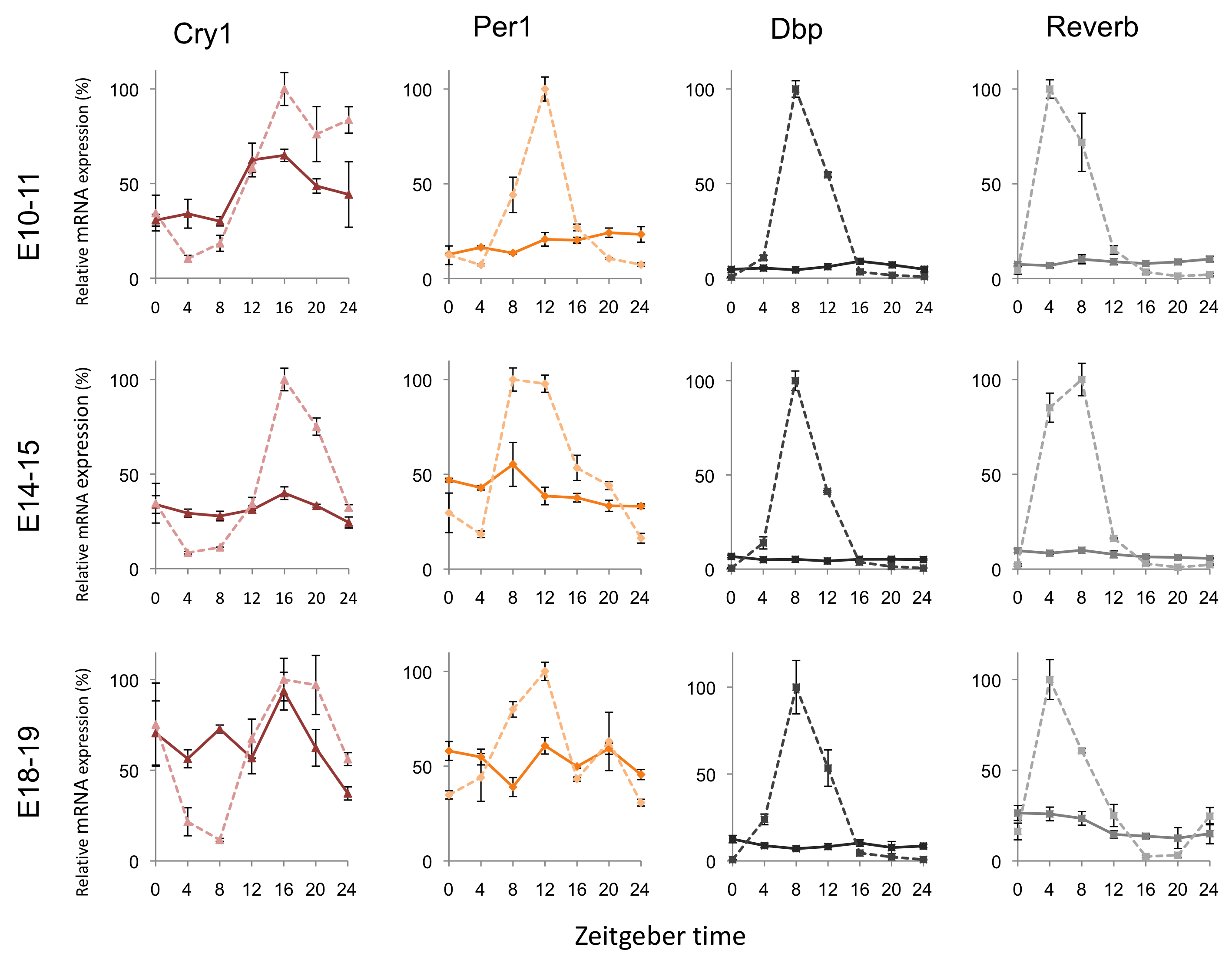

Supplement: Figure S1 — Twenty-four-hour expression profiles of Cry1, Per1, Dbp and Rev-erbα mRNA in whole embryos and maternal liver during embryogenesis measured by quantitative real-time RT-PCR. Whole embryos were collected every 4 hours for 24 hours on E10-E11 (A), E14-E15 (B) and E18-E19 (C) with the 0 and 24 hour time points representing repeated, independent measures of the same time of day. Maternal livers (broken lines) demonstrated robust (P<0.001) variation over 24 hours. With the exception of Dbp and Per1 at E10-E11, the embryonic mRNAs did not show significant variation at P = 0.01. RNA levels were normalized to the control gene, gapdh. Symbols represent the mean ± standard error of the mean (SEM) of three biological replicates. The maximum maternal liver RNA for each stage of gestation was set to 100 and the rest of maternal and embryonic samples are presented relative to that maximum. (0.82 MB TIF) [file pone.0009855.s001.tif]

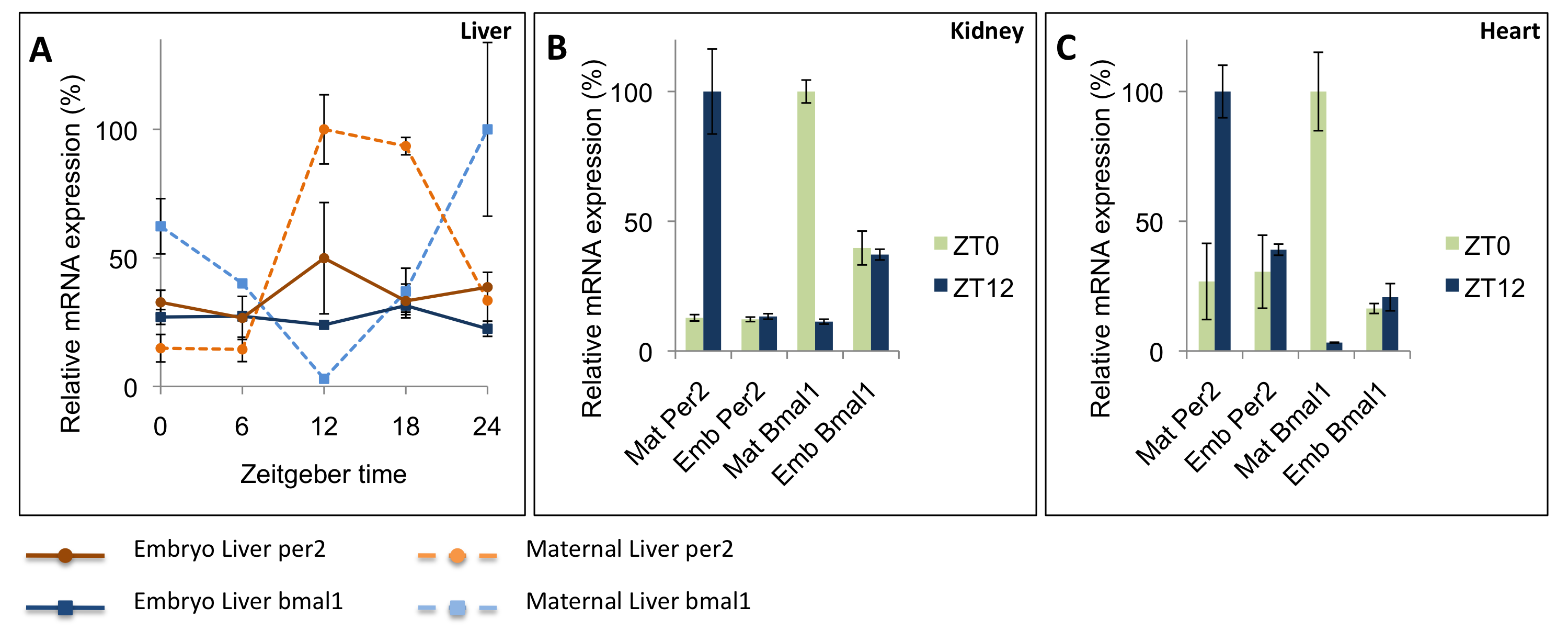

Supplement: Figure S2 — Expression of Per2 and Bmal1 mRNA in embryonic (E18-E19) and maternal tissues of melatonin positive mice (C3H). mRNA levels in embryonic liver (A) collected every 4 hours for 24 hours and measured using quantitative real-time RT-PCR showed low variation, especially in comparison to maternal liver. The maximum maternal liver mRNA was set to 100 and the rest of maternal and embryonic samples are presented relative to that maximum. In kidney and heart, levels of Per2 and Bmal1 mRNA from embryonic tissues were not significantly different at ZT0 and ZT12 (P>0.4, n = 3), while maternal tissues showed significant differences at these times (P<0.006, n = 3) (t-test). RNA levels were normalized to the control gene, gapdh. Error bars indicate standard error of the means. (0.59 MB TIF) [file pone.0009855.s002.tif]

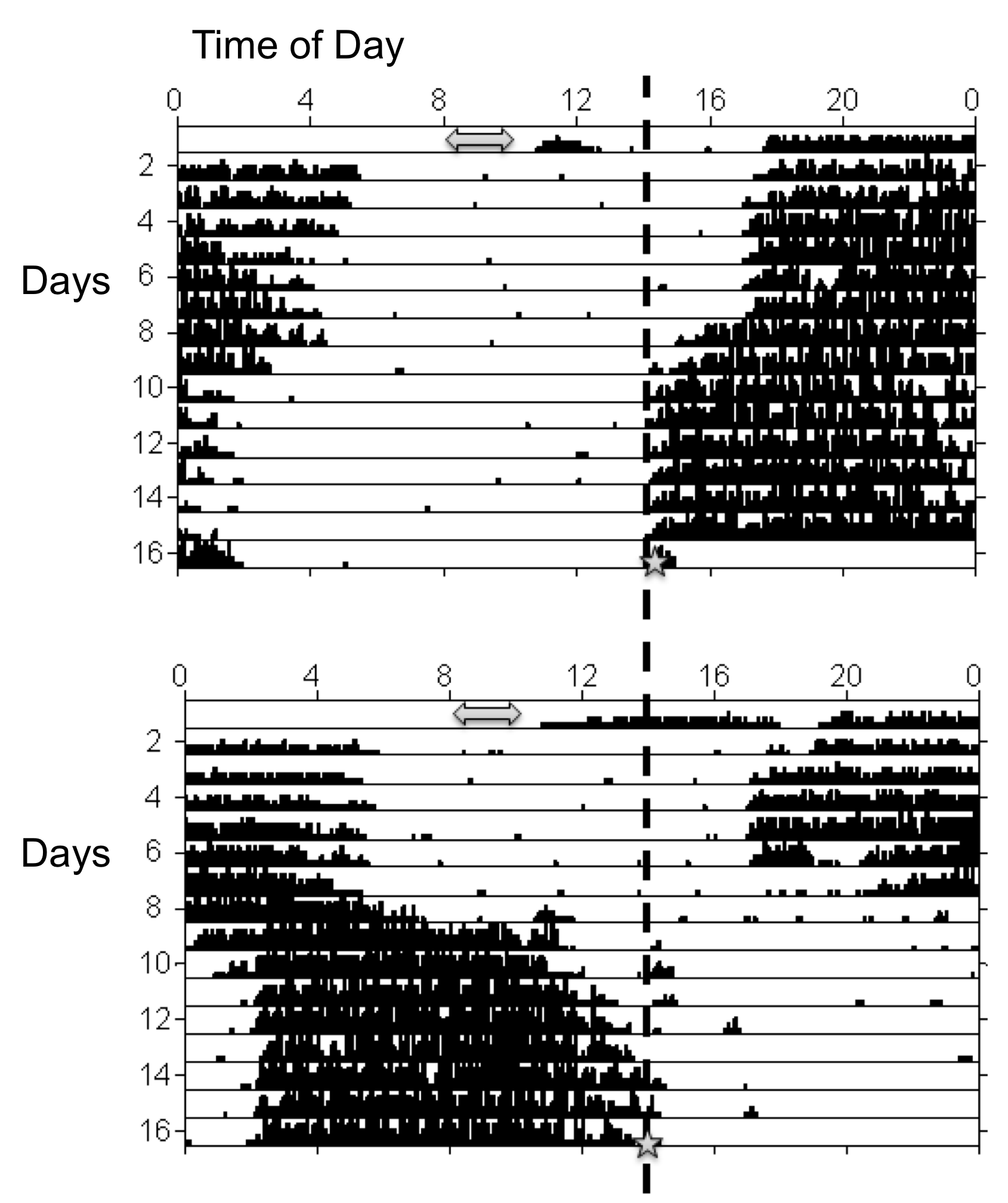

Supplement: Figure S3 — Entrainment of pregnant mice to opposite light:dark cycles. The wheel-running activity records of two pregnant mice are shown. Each line of the actograms is 24 hours of recording. Mice were paired with males just before recording was started (double arrow). Between days 5 and 7 of the records the light:dark cycles were shifted, an advance shift for the mouse on the top and a delay shift for the mouse on the bottom. When embryo tissues were collected on the last day of the records (star), the pregnant mice were fully entrained to opposite cycles. Thus the collection of embryo tissues occurred at one age but at two different times within the mothers' circadian cycles. (0.62 MB TIF) [file pone.0009855.s003.tif]
